# Soy Isoflavones Improve the Spermatogenic Defects in Diet-Induced Obesity Rats through Nrf2/HO-1 Pathway

**DOI:** 10.3390/molecules24162966

**Published:** 2019-08-15

**Authors:** Qihui Luo, Yifan Li, Chao Huang, Dongjing Cheng, Wenjing Ma, Yu Xia, Wentao Liu, Zhengli Chen

**Affiliations:** 1 Laboratory of Experimental Animal Disease Model, College of Veterinary Medicine, Sichuan Agricultural University, Chengdu 611130, China; 2 Key Laboratory of Animal Disease and Human Health of Sichuan Province, College of Veterinary Medicine, Sichuan Agricultural University, Chengdu 611130, China

**Keywords:** soy isoflavones, obesity, spermatogenesis, oxidative stress, apoptosis

## Abstract

Soy isoflavones (SIF) are biologically active compounds of non-steroidal and phenolic properties that are richly present in soybeans, which can reduce the body weight and blood lipids of obese animals. Recently, SIF have been reported to affect reproductive ability in obese male rats. However, the specific mechanism has not been well defined. The aim of the current study was to study the possible mechanisms for the effect of SIF administration on obesity induced spermatogenic defects. Obese rats model induced by high-fat diets were established and gavage treated with 0, 50,150 or 450 mg of SIF/kg body weight/day for 4 weeks. Here, our research shows that obesity resulted in spermatogenic degeneration, imbalance of reproductive hormone, testicular oxidative stress and germ cell apoptosis, whereas evidently recovery effects were observed at 150 and 450 mg/kg SIF. We also have discovered that 150 and 450 mg/kg SIF can activate Nrf2/HO-1 pathway in control of Bcl-2, BAX and cleaved caspase-3 expression with implications in antioxidant protection. Our study indicates the potential mechanism of SIF regulating spermatogenic function in obese rats, and provides a scientific experimental basis for the regulation of biological function of obese male reproductive system by SIF.

## 1. Introduction

Most developed countries have seen an unprecedented increase in obesity rates over the past few decades. In 2016, more than 1.9 billion adults were overweight and of these over 650 million were obese according to the World Health Organization (WHO) [1]. Obesity is a medical condition in which excessive fat accumulation has an adverse health effect. Body mass index (BMI) is usually used to define overweight (BMI, 25–29.9 kg/m^2^) and obesity (BMI ≥ 30 kg/m2) in adults [2,3]. The result from several studies suggested that obesity was an important risk factor for non-alcoholic fatty liver disease [4], hypertension [5], inflammation [6] and cancer [7]. Interestingly, data suggested obesity has a negative impact on male reproductive system and fertility over the past decade [8,9,10,11].

It has been reported that systemic oxidative stress induced by obesity [12] not only damages the integrity of sperm deoxyribonucleotide (DNA) [13] but also restricts the fertilizing potential of these cells via collateral damage to proteins and lipids in the sperm plasma membrane [14,15]. Oxidative stress happens owing to excessive ROS production and poor ROS scavenging system efficiency, whereby free radicals are indispensable for cellular processes, however, at increased concentrations, can also interfere with essential metabolic processes [16]. Thus, obesity changes the function of the male reproductive, which is closely related to oxidative stress (OS) [17]. Nrf2 (NF-E2-related factor 2) is a cytoprotective regulator of the antioxidant defense gene and is involved in signal transduction in the regulation of gene expression mediated by antioxidant response elements (ARE). Under oxidative stress conditions, the Nrf2 accumulates in the nucleus binds to the ARE promoter and up-regulates a range of antioxidant and cytoprotective genes, including superoxide dismutase (SOD), catalase, glutathion sulfhydryl transferase (GST), glutathione peroxidase (GSH-Px), hemeoxygenase-1 (HO-1) and NAD(P)H-quinone oxidoreductase 1 (NQO-1). These genes play a protective role in antioxidant defense. Meanwhile, a previous report of Nrf2 as a promising drug target to treat obesity speculated that Nrf2 may be the key factor of antioxidant reaction in testes [18,19]. In fact, oxidation and antioxidant states in the testis are usually balanced, which is beneficial to normal spermatogenesis. Nrf2/HO-1 is the key regulatory factor of oxidation-reduction process, but obesity breaks down the redox state in the testis, making the expression of Nrf2/HO-1 abnormal, resulting in testicular oxidative stress and affecting spermatogenesis.

The hypothalamic-pituitary-testis axis regulates the process of spermatogenesis, and FSH directly binds to receptors on testicular Sertoli cells that can produce androgen-binding proteins (ABP). Dihydrotestosterone (DHT) is an androgen produced by the reaction of testosterone with 5 α-reductase. ABP brings androgens to germ cells and dissociates from androgens. High concentrations of androgens promote the development of sperm [20]. In addition, the number of sperm is positively correlated with testosterone levels. A significant decrease in intratesticular testosterone concentration appears to be an important trigger for apoptosis in spermatogenic epithelial cells [21,22]. In conclusion, male obesity is associated with decreased sperm density and viability, increased sperm DNA damage and changes in reproductive hormones. One of the underlying pathological mechanisms behind the decline in reproductive capacity in obese men is sperm oxidative stress [23].

Obviously, it is urgent to control the occurrence of obesity and its complications. Studies found that the low incidence of obesity related diseases in Asian countries is related to the high consumption of isoflavones-containing soybeans. Therefore, the research focus of scholars turns to the biological function of soybean, among them, the most widely studied is soy isoflavones (SIF) [24,25,26]. SIF are mainly natural active substances produced by legumes and soybeans are the richest sources of SIF in the human diet [27]. The chemical structure of SIF is similar to the 17β-estradiol (E2), and shows anti-oestrogenic activity by competing with the endogenous estrogen for estrogen receptor (ER) binding, so it is also called phytoestrogen [28,29]. The results from in vitro and in vivo investigations have proved the effects of SIF on tumor suppression, however, it has become obvious that the mechanism of function may not be only hormonal [30]. In addition, some scholars have also found that SIF can effectively improves plasma lipoproteins and, consequently, may attenuate risk of coronary heart disease [28]. Several studies suggest that dietary SIF exhibit a positive effect on antioxidant status, and enhances antioxidant level of plasma and antioxidant enzyme activity in multiple tissues [29,31,32].

However, when SIF were utilized to achieve lipid-lowering and weight-reducing effects, few studies have focused on the impact of SIF on the obese male reproduction. One experiment on obese male rat demonstrated that SIF significantly improved the expression of proteins and genes related to testosterone synthesis [33], but the effect of the SIF on spermatogenic ability and its mechanism is unclear. In the study, we explored whether SIF may exert antioxidation to improve spermatogenic defects. Therefore, we established diet-induced obese (DIO) rat model and then introduced supplementation with different doses of SIF to explore SIF-induced changes and potential mechanism of action on testicular spermatogenesis in obese rats.

## 2. Results

### 2.1. SIF Promote Spermatogenesis in DIO Male Rats

**Figure 1:**
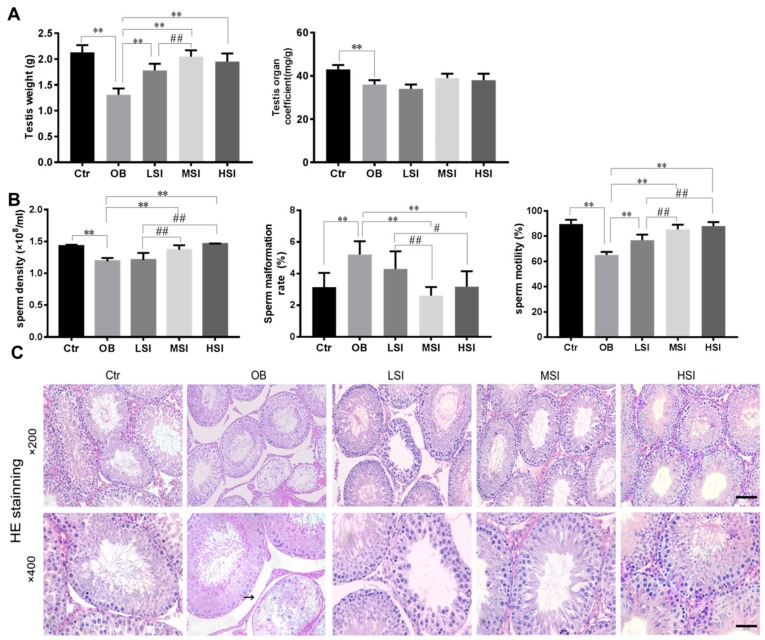

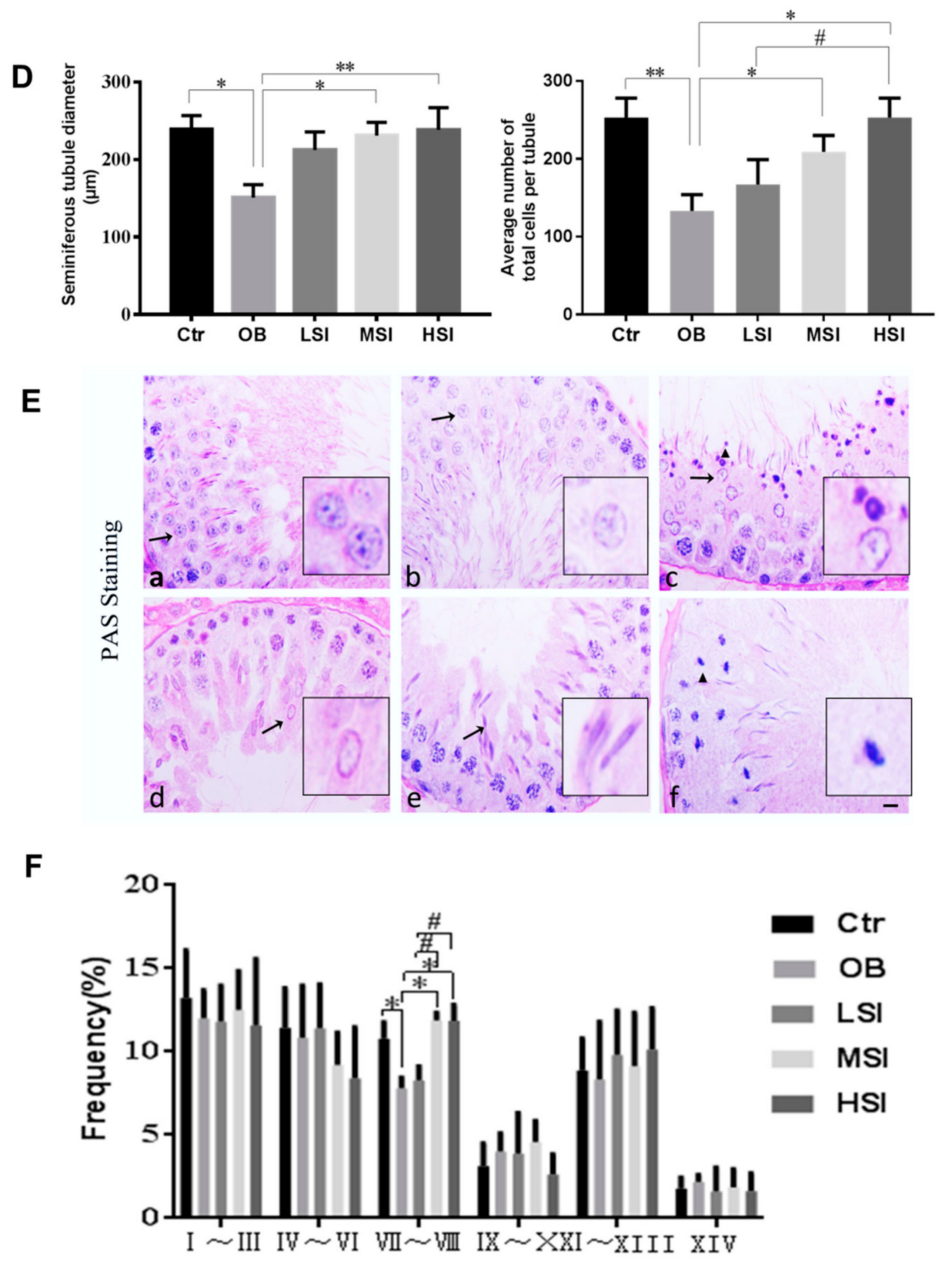
Soy isoflavones (SIF) promote spermatogenesis in diet-induced obese (DIO) male rats. (**A**) Effects of SIF on the testis weight and the testicular coefficient of adult male obese rats. (**B**) Effects of SIF on the sperm parameters of adult male obese rats. (**C**) Histological morphology of testis stained with HE from the five groups; pathological changes of seminiferous tubule (black arrow); magnification: ×200; scale bar: 100 μm. Magnification: ×400; scale bar: 50 μm. (**D**) Quantifcation shows the diameter of seminiferous tubules and mean number of the total cells per tubule of the five groups. (**E**) The testicles of rats staining with PAS-H staining. (a) I–III stages. Acrosomal vacuole (black arrow); (b) IV–VI stages. Acrosome (black arrow); (c) VII-VIII stages. Acrosome (black arrow) and residual body (black triangle); (d) IX-X stages. The circular sperm nuclei move from the center to the cell near the cell membrane side (black arrow); (e) XI-XIII stages. Sperm cells are further deformed, nuclear condensation(black arrow); (f) XIV stages. Meiotic phase (black triangle). Scale bar: 10 μm; Magnification: ×1000. (**F**) Quantification shows the seminiferous epithelial cycle count. Each bar denotes mean ± SD of eight rats. * *p* < 0.05, ** *p* < 0.01 vs. OB group; # *p* < 0.05, ## *p* < 0.01 vs. LSI; Δ *p* < 0.05, ΔΔ *p* < 0.05vs. MSI, one-way ANOVA analysis and Least—Significant Difference (LSD) test.

Sperm is produced in the testis and stored in the epididymis, so we observed the morphological structure of the testicular spermatogenic epithelium and the epididymal sperm parameters. In our studies, obesity induced by high-fat diets significantly reduced testis weight and testis organ coefficient in rats compared with the control group (during the SIF intervention, the basic diet group was also called the control group, Ctr). After SIF intervention, testis weight was significantly higher compared with the obesity group (OB). However, dietary SIF failed to affect weight coefficient of testis in obese rats (Figure 1A). It is well known that sperm concentration, motility and morphology are the most intuitive ways to assess male fertility. The changes of sperm parameters in obese rats treated with SIF are summarized in Figure 1B. Sperm density in obese rats was significantly increased in response to treatment with 150 or 450 mg/kg SIF. Obese rats had a higher abnormal sperm morphology ratio than that of the Ctr group, but 150 mg/kg SIF (medium-does SIF, MSI) or 450 mg/kg SIF (high-does SIF, HSI) significantly cut down the abnormal sperm morphology ratio in obese rats. Furthermore, obese rats had lower sperm motility than that of the Ctr group, and after the intervention of SIF, the effect of obesity was reversed. Hematoxylin-eosin (HE) staining showed that the seminiferous tubules became deformed or atrophied, and cell adhesions between spermatogenic cells and Sertoli cells were loosely arranged in testicles of obese rats. No obvious histological damage was observed following treatment with 150 and 450 mg/kg SIF (Figure 1C). Meanwhile, the MSI and HSI groups exhibited normal tubule diameter and number of cells in each seminiferous tubule (Figure 1D). To explore the impact of SIF on spermatogenic cycle in obese rats, we further performed the periodic acid-Schiff-hematoxylin (PAS-H) staining to observe the changes in acrosome formation of round spermatids during the course of multistage spermatogenesis (Figure 1E). Statistical analysis of the seminiferous epithelium phase showed that the changes were mainly concentrated in phases VII, VIII (Figure 1F). Compared with the Ctr group, the VII and VIII phases of the OB group were significantly lower. For obese rats, significant increase in phases VII and VIII were observed following 150 and 450 mg/kg SIF administration. However, there was no significant difference between the 50 mg/kg SIF (low-does SIF, LSI ) group and the OB group.

### 2.2. Effects of SIF on Plasma Reproductive Hormone in DIO Male Rats

**Figure 2:**
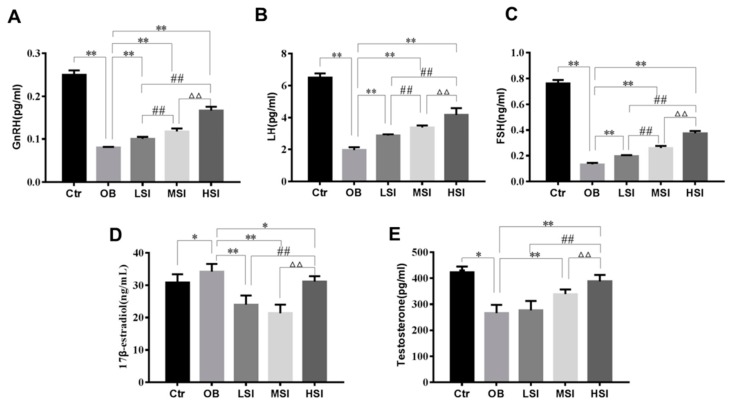
Effects of SIF on plasma reproductive hormone. Quantification shows (**A**) plasma gonadotropin-releasing hormone (GnRH) levels, (**B**) plasma luteinizing hormone (LH) levels, (**C**) plasma follicle-stimulating hormone (FSH) levels, (**D**) plasma E2 levels and (**E**) plasma T levels. Bars represent the mean ± SD, * *p* < 0.05, ** *p* < 0.01 vs. OB; # *p* < 0.05, ## *p* < 0.01 vs. LSI; Δ *p* < 0.05, ΔΔ *p* < 0.05 vs. MSI, one-way ANOVA analysis and LSD test.

Testosterone (T) has a pivotal role on the beginning and maintaining of spermatogenesis, and luteinizing hormone (LH) plays a key role in the production of testosterone by stimulating Leydig cells. Sertoli cells are the somatic cells within testis that are regulated by follicule-stimulating hormone (FSH) stimulation and paracrine function of T to support spermatogenesis [34]. The levels of plasma FSH, LH and gonadotropin-releasing hormone (GnRH) in the OB group were significantly lower than those in the Ctr group, and reached the minimum. It was worth noting that plasma FSH, LH and GnRH concentrations in SIF groups were markedly enhanced relative to the obesity group and also showed a dose-dependent increase (Figure 2A–C). In obese rats, the plasma T level was remarkably decreased, while plasma 17β-estradiol (E2) level was significantly increased compared with the control. SIF significantly decreased the plasma E2 level when compared to obese rats. The T level among the 150 and 450 mg/kg SIF-added groups were significantly different from those in OB group. More interestingly, the 450 mg/kg SIF was significantly better than the 150 and 50 mg/kg SIF in the abnormal recovery of plasma T and E2 levels in obese rats (Figure 2D,E).

### 2.3. Effects of SIF on Testis Oxidative Stress in DIO Male Rats

**Figure 3:**
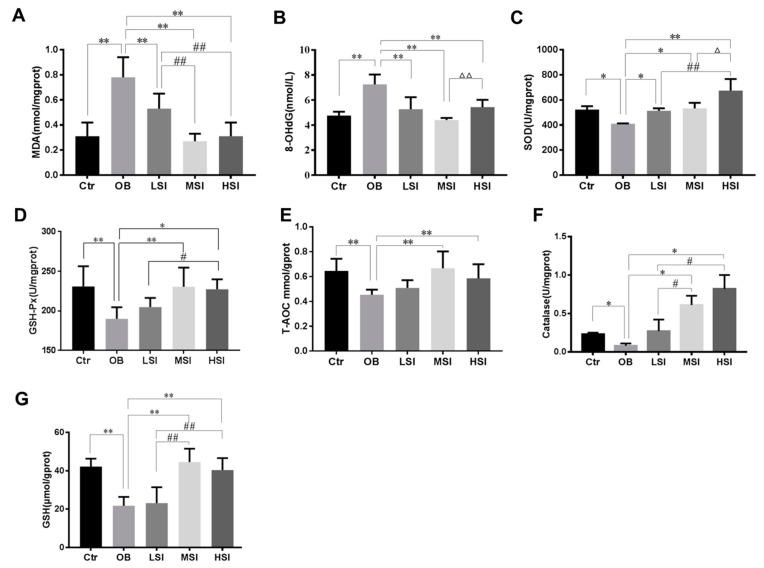
SIF treatment attenuated oxidative stress. (**A**–**G**) Effect of SIF on malondialdehyde (MDA), 8-OhdG, superoxide dismutase (SOD), glutathione peroxidase (GSH-Px), total anti-oxidant capacity (T-AOC), catalase (CAT) and reduced glutathione (GSH) of testicular tissue in rats (*n* = 8). Bars represent the mean ± SD, * *p* < 0.05, ** *p* < 0.01 vs. OB; # *p* < 0.05, ## *p* < 0.01 vs. LSI; Δ *p* < 0.05, ΔΔ *p* < 0.05 vs. MSI, one-way ANOVA analysis and LSD test.

**Figure 4:**
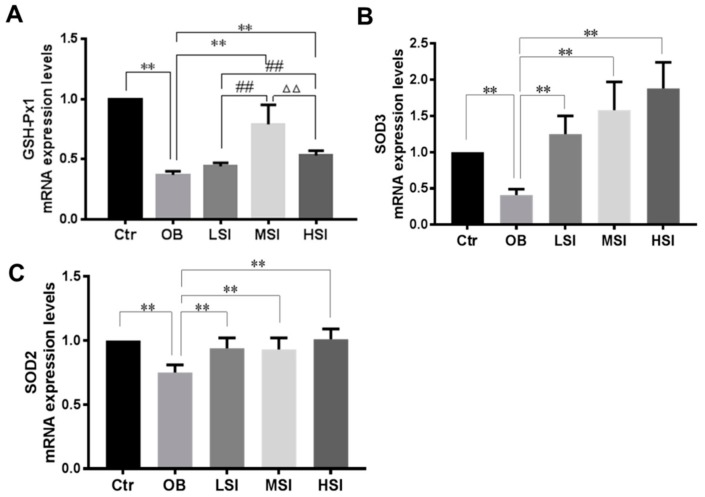
Effect of SIF on mRNA expression level of (**A**) GSH-Px1, (**B**) SOD3, (**C**) SOD2 (*n* = 8). Bars represent the mean ± SD, * *p* < 0.05, ** *p* < 0.01 vs. OB; # *p* < 0.05, ## *p* < 0.01 vs. LSI; Δ *p* < 0.05, ΔΔ *p* < 0.05 vs. MSI, one-way ANOVA analysis and LSD test.

**Figure 5:**
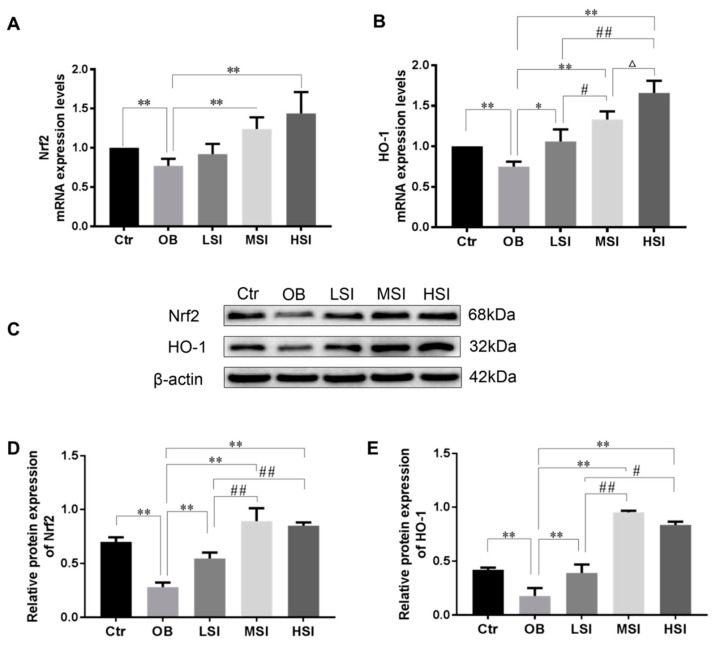
SIF treatment resulted in the activation of Nrf2/hemeoxygenase 1 (HO-1). (**A**,**B**) represent mRNA expression level of Nrf2 and HO-1 in the indicated groups (*n* = 8). (**C**) represents western blotting (WB) banding pictures of Nrf2 and HO-1. (**D**,**E**) represent protein expression levels of Nrf2/HO-1 in the testis tissue of the indicated groups (*n* = 8). Bars represent the mean ± SD, * *p* < 0.05, ** *p* < 0.01 vs. OB; # *p* < 0.05, ## *p* < 0.01 vs. LSI; Δ *p* < 0.05, ΔΔ *p* < 0.05 vs. MSI, one-way ANOVA analysis and LSD test.

It has been demonstrated that intratesticular oxidative stress was implicated in obesity induced spermatogenic defects [35,36]. The testis malondialdehyde (MDA) and 8-hydroxy-2 deoxyguanosine (8-OHdG) contents, as markers of lipid peroxidation and DNA oxidation damage respectively, elevated significantly in obese rats compared to the Ctr group, while these parameters were significantly decreased after SIF administrated (Figure 3A,B). Our results showed that the obesity induced significant reductions of intratesticular SOD, catalase, GSH-Px activities and total anti-oxidant capacity (T-AOC) level, and the content of reduced glutathione (GSH) compared with the Ctr group. SIF significantly increased the activities of SOD in the testis of obese rats (Figure 3C–G). Compared with the OB group, the T-AOC level, CAT and GSH-Px activity, and GSH content of the LSI group did not alter significantly, but 150 and 450 mg/kg SIF significantly increased these parameters (Figure 3D–G). Similarly, mRNA levels of SOD2, SOD3 and GSH-Px1 were significantly increased in obese rats with SIF treatment as compared to the OB group (Figure 4). Gene and protein expressions of Nrf2 and HO-1 are exhibited in Figure 5. Compared to the Ctr group, the genes and protein expression levels of Nrf2 and HO-1 were visibly decreased in the OB group. Amounts of 150 and 450 mg/kg SIF significantly increased Nrf2 and Bcl-2 mRNA transcription levels in obese rats but not following treatment at the 50 mg/kg SIF. The mRNA level of testicular HO-1 was significantly up-regulated in obese rats treated with SIF in a dose-dependent manner, and the exposure of SIF increased Nrf2 and HO-1 expressions compared with the obese rats, which suggested that SIF may exert its protective effects in obese rat testis oxidative injury through Nrf2/HO-1 pathway.

### 2.4. Effects of SIF on the Testis Cell Apoptosis in DIO Male Rats

**Figure 6:**
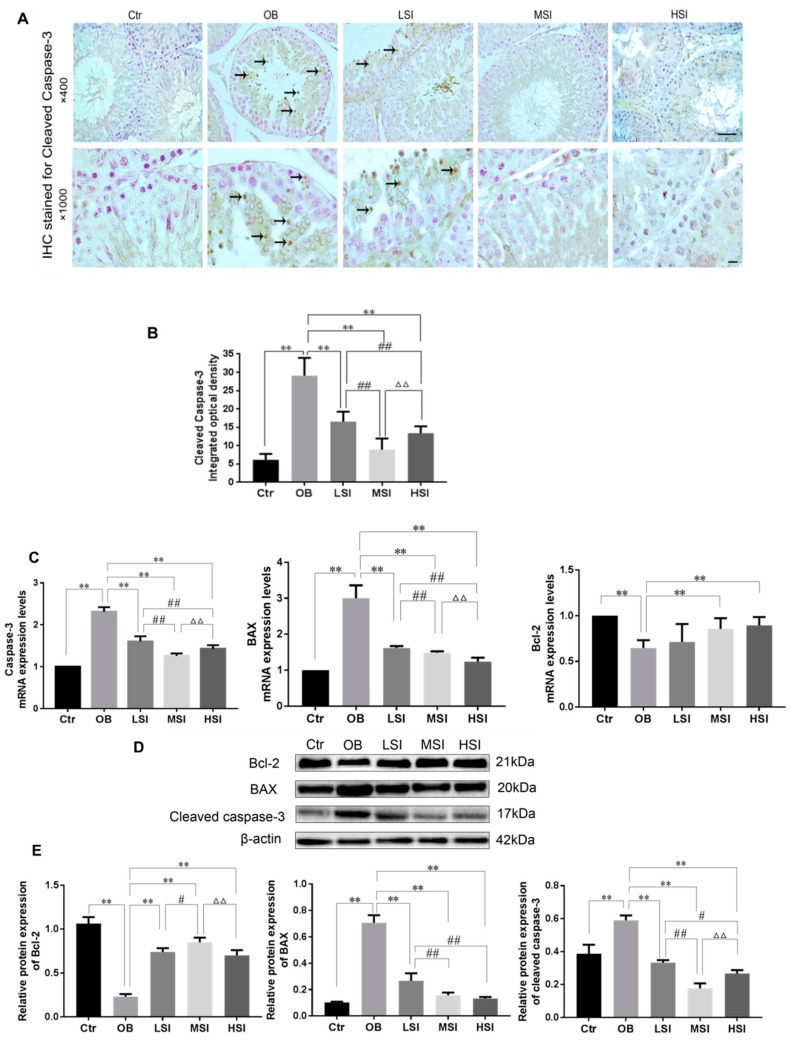
SIF reduced excessive apoptosis of testicular germ cells in obese rats by increased levels of Bcl-2 and decreased levels of BAX and caspase-3. (**A**) IHC detection of cleaved caspase-3 expression in testicular tissue. Cleaved caspase-3 positive and negative cells were stained in brown colored and bluish violet, respectively. Black arrowheads: Cleaved caspase-3 positive cell. Magnification: 400×; scale bar: 50 μm. Magnification: 1000×; scale bar: 10 μm. (**B**) IOD of cleaved caspase-3 protein expression in the testis. (**C**) mRNA expression level of Bcl-2,BAX and caspase-3 in the indicated groups (*n* = 8). (**D**,**E**) proteins expression level of Bcl-2, BAX and cleaved caspase-3 in the indicated groups (*n* = 8). Bars represent the mean ± SD, * *p* < 0.05, ** *p* < 0.01 vs. OB; # *p* < 0.05, ## *p* < 0.01 vs. LSI; Δ *p* < 0.05, ΔΔ *p* < 0.05 vs. MSI, one-way ANOVA analysis and LSD test.

Many studies have shown that oxidative stress in testes can induce apoptosis of germ cells [37,38]. Hence, we observed the apoptosis of testicular germ cells by immunohistochemistry staining (IHC) of cleaved caspase-3 protein. High intensity of immunostaining for cleaved caspase-3 was observed in the testis of obese rats as compared to the Ctr group. Cleaved caspase-3 protein was distributed in round sperm cells and spermatogonia in testis of obese rats (Figure 6A). There was a significant reduction in integral optical density (IOD) of cleaved caspase-3 in the testes of SIF supplement rats compared to the OB group. In addition, the positive expression of cleaved caspase-3 in the MSI group was significantly lower than that in the LSI and HSI group (Figure 6B). Then we also detected the expressions of key factors controlling apoptosis. Gene expression level of BAX, Bcl-2 and caspase-3 of each group is exhibited in Fig 6C. In the obese rats, compared to the control rats, the levels of BAX and caspase-3 mRNA were significantly increased, while the expression levels of Bcl-2 were visibly decreased. Caspase-3 and BAX, the markers of cell apoptosis, were down-regulated by SIF compared with the OB group, especially for BAX mRNA level, in a dose-dependent reduction (Figure 6C). As shown in Figure 6D,E, at the protein levels, quantifications of the three proteins indicated that the expressions of both cleaved caspase-3 and BAX reached the maximum at the OB group, while expression of Bcl-2 decreased in the OB group compared with the Ctr group. Consistent with the alteration mRNA level of caspase-3 above, the testis protein levels of cleaved caspase-3 significantly decreased after treatment with SIF in obese rats as shown by western blot and the quantification analysis. Similarly, as compared to the OB group, significant decrease of BAX protein expression was observed in the SIF-added groups.

## 3. Discussion

Worldwide epidemiological analysis has indicated that soy isoflavones intakes may attenuate obesity [39]. Recently, several studies have reported sperm concentration and deformity rate were negatively correlated with obesity as defined by body mass index (BMI) [40,41]. Similarly, in animals, obesity has an influence on spermatid formation, resulting in a reduced sperm count [42]. Undoubtedly, obesity can lead to low spermatogenic function and even infertility in males, but it is necessary to understand the impacts on spermatogenesis when a SIF-supplemented diet is used for obese men. For the absence of information on this subject, we investigated the effects of SIF on testis weight, testis/body weight, epididymal sperm parameters, and testicular microstructure in obese rats. The doses of SIF selected in this study make a slightly modified reference to the guidance of Huang et al [33,43]. Under our experimental conditions, a direct relationship between SIF exposure and the degree of recovery in epididymal sperm parameters in obese rats was observed. In the present study, SIF significantly increased sperm motility in obese rats. Importantly, only medium- and high-doses SIF can improve low sperm density in obese rats. On the basis of previous studies, the structure of acrosome in the spermatid as confirmed by periodic acid-Schiff staining is used to divide the stages of the cycle of the seminiferous epithelium [44,45,46]. In our study, the Ⅶ~Ⅷ stages of seminiferous epithelium with medium- and high-does SIF groups were considerably increased as compared to the obesity group, and the histopathological analyses highlighted seminiferous tubule recovery after the same does of SIF exposure compared with the obesity rats. Our results suggest that increases in soy isoflavones uptake can protect from obesity-induced spermatogenic abnormalities in adult rats, particularly 150 and 450 mg/kg soy isoflavones. Testicular oxidative stress appears to be one of the main causes of male infertility, which can lead to dynamic changes in testicular microvascular blood flow, endocrine signals, and germ cell apoptosis [47]. Mammalian spermatozoa membranes contain large amounts of polyunsaturated fatty acids, making them consequently susceptible to lipid peroxidation [10]. In addition, spermatogenesis is a continuous process of proliferation and differentiation, and the mitochondria of the germinal epithelium consumes a high amount of oxygen, accompanied by the production of reactive oxygen species. This result may be exacerbated by fat accumulation, which is due to an increase in fatty acid supply owing to increased fat intake in the testicular environment, so more fatty acids enter the mitochondria and are oxidized [48]. Studies have shown that SIF have the ability to scavenge free radicals, reduce the sensitivity of DNA to oxidative stress, and increase the activity and expression of antioxidant enzymes [31]. Similarly, Liu et al. reported that soy isoflavones (150 and 200 ppm) had marked increases in SOD and CAT activities in vitro and in vivo. The content of MDA, 8-OhdG, T-AOC and GSH, and anti-oxidase activities can reflect the degree of oxidative stress [49,50,51,52]. Indeed, in the present study, the SIF treatment resulted in a significant increase in SOD, GSH-Px, and CAT activities, the content of GSH, and T-AOC levels with decreasing content of MDA and 8-OHdG in testicular tissue compared with the obese rats, enhanced the antioxidant defense system. It’s worth mentioning that between the low-does SIF group and the obesity group, that we witnessed no significant change in GSH-Px, and CAT activities, the content of GSH, and T-AOC levels. Likewise, SIF increased testicular antioxidant-related genes mRNA expression (i.e., GH-Px1, SOD2 and SOD3) in obese rats. Hence, it is logical to expect that the mechanism of SIF in improving spermatogenesis anomaly in the testis of obese rats is due to the elimination or reduction of oxidative damage.

Apoptosis is a natural event that regulates germ cell turnover and maintains spermatogenesis [53]. Excessive apoptosis of spermatogenic cells would damage spermatogenesis leading to infertility [54]. Increased evidence indicates that altering testis morphological structure and increases in germ cell apoptosis were present in high-fat diet induced obesity or lipid metabolism disorder [55]. Further, testosterone provides protection against apoptosis of germ cell [56]. Cleaved caspase-3 is one of the crucial implementers of apoptosis [57]. To substantiate the mechanism of cell death, immunohistochemical staining of cleaved caspase-3, an indicator of cell apoptosis in the testicular tissue assays, was carried out. The present study revealed that obesity increased cleaved caspase-3 positive immunostaining in the testicular tissue than the control group. Similarly, Jia Y.-F. et al. showed that the apoptosis of testicular spermatogenic cells with TUNEL staining obviously increased in obese rats [58]. In the current study, immunohistochemical staining had shown decreased expression of cleaved caspase-3 in testicular cells of SIF-treated rats in comparison to the OB group. All those effects indicate that obese rats’ exposure to a medium or high dose of SIF may significantly recover redox state, ultimately contributing to increased sperm motility and sperm production, and decreases in the teratospermia rate. It has been proven that estrogen plays an important role in various spermatogenesis processes [34]. Obesity caused by overconsumption of high-fat diet increases the whole-body levels of E2, due to increased activity of aromatase in the adipocytes. The increase in systemic levels of E2 acts on the hypothalamus-pituitary via negative feedback, thereby restraining GnRH and pituitary gonadotropins [59]. Since SIF has a biological activity binding to the estrogen receptor [60], we studied its impact on plasmatic reproductive hormone regulated by the hypothalamus–pituitary–testicle (HPT). On one hand, in present study, obesity induced by high-fat diet down-regulated plasma GnRH, FSH, LH, and T levels, while it up-regulated E2 levels compared to the control group, as Vigueras-Villaseñor R M et al. [61] reported in a similar experiment. In addition, studies have shown that T is required for the conversion of the seventh step of spermatids into the eighth step of spermatids [62,63,64], and that the step of spermatids seventh to the eighth are in the Ⅶ~Ⅷ stages of the spermatogenic epithelial cycle. On the other hand, we demonstrated that SIF abated reproductive endocrine abnormalities associated with obesity, including the plasma levels of GnRH, gonadotropin, T and E2. The changes of reproductive hormone level were highly consistent in the recovery of related markers of spermatogenesis (i.e., germ cell number, sperm density and cycle of seminiferous epithelium) in obese rats by SIF exposure.

SIF are the main active ingredients in soybeans and possess a high antioxidant activity [65]. Several researches have shown SIF administration can activate Nrf2-mediated antioxidant responses [65,66,67,68]. Nrf2 induces expression of HO-1, an enzyme that catalyzes the degradation of heme into carbon monoxide (CO) and free iron (Fe), and biliverdin to bilirubin. CO and bilirubin possess anti-oxidant activities. Increased expression of HO-1 provides protection against OS [69]. In addition, Nrf2 disturbs apoptosis-related events through up-regulating Bcl-2, down-regulating BAX and caspase-3 [70,71]. In the present study, we examined the expression of related genes and proteins during testicular antioxidant defense and apoptosis. In the testis of obese rats, Nrf2, HO-1 and Bcl-2 expression were down-regulated, whereas caspase-3 and BAX expression were upregulated. By contrast, exposure to SIF, particularly at medium or high doses, attenuated these effects.

Moreover, SIF can also be considered as endocrine disruptors, which may have a negative impact on the health of the fetal period. The fetal development is one of the most sensitive periods in human life [72]. Owing to the mother’s lifestyle (e.g., vegetarian diet, food supplement or soy milk consumption), prenatal exposure may occur when isoflavones cross the placental barrier and enter the fetal circulation [73]. AbdelaliLehraiki et al. reported that genistein impairs early testosterone production in fetal mouse testis [74]. To prove that hormonal imbalance in experimental rats from perinatal exposure to genistein (Gen) may also cause detrimental effects on the morphology of the testis in adulthood. Nurul Iftitah Musameh et al. from the 10th day of pregnancy in SD rats, administered Gen 1, 10 and 100 mg/kg body weight doses for 5 weeks, and the result showed that diet containing Gen10 and Gen100 effectively stimulate spermatogenesis in rat testes as evidenced by a significant increase in germinal epithelial thickness [75]. Although our results mainly show the beneficial effect of SIF on the reproductive ability of obese rats, the effect of SIF has been controversial, especially on function of fetal testicles.

In conclusion, this study shows that SIF treatment balances the redox state of testis tissue and ameliorates reproductive endocrine-disrupting effects caused by obesity and subsequent promote anti-oxidative function in obese rats to spermatogenesis. Furthermore, the results suggest that SIF induces the expression of anti-apoptotic proteins, and reduces the expression of pro-apoptosis proteins, and all of these may be done through the Nrf2/HO-1 pathway. These findings contribute to understanding the mechanism of SIF on obese male fertility. Further studies are needed to explore the accurate signaling pathways by which SIF protects testis from obesity-induced dysfunction.

## 4. Materials and Methods

### 4.1. Animals Care, Diets and Experimental Design

Forty 5-week-old male healthy Sprague Dawley (SD) rats weighing 180~220 g were purchased from Dashuo (Chengdu, Sichuan, China) (license number: SCXK [Sichuan] 2015-030; license number: SYXK [Sichuan] 2014-187). All animals were housed under standard laboratory conditions with a 12-h light/dark cycle at constant temperature (22 ± 2 °C) and humidity (55 ± 5%) and kept on basal diet and tap water provided ad libitum. The animal protocols and all procedures of the experiment were performed in compliance with the laws and guidelines of Sichuan Agricultural University Animal Care and Use Committee, and the study was approved by The Ethics Committee for Animal Experiments (Institute of Animal Diseases and Environmental Hazards of Sichuan Province, Chengdu, China). After a one-week acclimatization period, they were randomly divided into two groups, namely the BD group (*n* = 8, continuously fed with basic diet for further 12 weeks) and the HFD group (*n* = 32, continuously fed with high fat diet for further 12 weeks). The basic diet contained 54% corn, 14% wheat bran, 13% alfalfa meal, 10% cotton meal, 6% fish meal, 1.5% vitamin and mineral, 1% limestone, 0.3% sodium chloride and 0.2% dicalcium phosphate. High-fat diet (69.5% basic diet, 15% pork fat, 15% sucrose and 0.5% pig bile) were purchased from Primed (Ya’an, Sichuan, China). The rats were weighed once a week and the obesity model is considered successful when the average body weight of the HFD group is 1.4 times than the BD group. Then, the high-fat diet rats were randomly divided into four groups (*n* = 8 per group), and were administered SIF dissolved in 0.5% carboxymethylcellulose sodium (CMC-Na) by gavage at 0, 50, 150 and 450 mg/kg body weight/day starting from ninth week for 28 days. At the same time, rats from the Ctr group and the OB group (0 mg/kg body weight SIF) were gavage with 0.5%CMC (2 mL/kg). Five groups of rats were weighed accurately and recorded weekly, and the weight of testis were measured at the end of the whole experiment. After SIF treatment for 4 weeks, all the rats were anesthetized with10% chloral hydrate (2 mL/kg body weight).

### 4.2. Collection of Biological Samples

Rats’ blood was collected from the lateral tail vein. The blood was allowed to coagulate for 2 h at 4 °C and then centrifuged at 3000 rpm for 10 min. Serum was separated and stored at −20 °C until biochemical analysis. The testis were dissected out immediately, trimmed off the attached tissues and weighed on the ice plate. The cauda epididymis was used for sperm analysis. The left testis were wrapped in tinfoil and frozen in liquid nitrogen and stored at −80 °C for protein and nucleic acid extraction. The right testis were fixed with modified Davidson’s fluid for haematoxylin-eosin (H&E) staining and immunohistochemistry.

### 4.3. Sperm Count, Motility, and Morphology

The cauda epididymides were dissected out immediately. Then, epididymides were rinsed briefly in pre-warmed physiological saline (37 °C) and made sperm suspension in 1 mL of sperm nutrient solution (0.35 g/L NaHCO3, 4.2 g/L HEPES, 2.0 g/L BSA, 0.35 g/L NaHCO3, 0.1 g/L sodium pyruvate, 0.9 g/L D-glucose, and 0.025 g/L soybean trypsin inhibitor in Hanks balanced salt solution, pH = 7.4, 37 °C). A drop of sperm suspension was placed on a hemocytometer and progressive (PR), non-progressive (NP), immotility (IM) and total sperm were counted according to the WHO guidelines. For determination of sperm malformation rate, a drop of the suspension was placed on a clean slide and smeared evenly, after which the smears were air dried, fixed with 1% paraformaldehyde, and then stained with 2% eosin for 1 h. The slides were washed in water and air dried again. The final sperm concentration were expressed as 106/mL sperm suspension, the final sperm motility and deformity rate were expressed as a percentage of PR + NR and deformed sperm in the total number of spermatozoa, respectively. For each individual, at least 300 sperm were examined at ×400 magnification.

### 4.4. Histological Analysis and Staging of Seminiferous Epithelium

Fixed testes were cut into the proper size embedded in paraffin and serially sectioned (5 μm) with stained HE and PAS-H. The stained results were observable using a Leica light microscopy (×200, ×400 or ×1000 magnification). 100 seminiferous tubules were observed in each section at a magnification of ×400. Seminiferous tubules were chosen according to the same criterion. A histogram of the number of germ cells in 100 seminiferous tubules was analyzed.

### 4.5. Hormone Assays

According to the standard protocol supplied by the manufacturer, we determined the plasma concentration of gonadotropin-releasing hormone (GnRH, JYM0426Ra), follicule-stimulating hormone (FSH, JYM0859Ra), luteinizing hormone (LH, JYM0623Ra), 17β-estradiol (E2, JYM0608Ra) and testosterone (T, JYM0610Ra) with commercial enzyme-linked immunosorbent assays (ELISA) kit (Wuhan Colorful-Gene biological technology Co., Ltd., China). All samples and standards were run in triplicate.

### 4.6. Oxidative Stress Markers in the Testis

Testis tissue (100 mg) was homogenized in 0.9 mL ice-cold 0.86% saline buffer (1:9, weight/volume) using an JY92-IIN ultrasonic cell disruptor (Xinzhi biotechnology co., Ltd., China) and then centrifuged at 3000 r for 10~15 min at 4 °C to obtain supernatants at a concentration of 0.1 g/mL for further testicular redox state. 8-OHdG (Shanghai Enzyme-linked Biotechnology Co., Ltd, Shanghai, China), MDA, SOD, GSH-Px were determined with clinical chemistry assay kits (Nanjing Jiancheng Bioengineering Institute, Nanjing, China) according to the manufacturer’s instructions.

### 4.7. Immunohistochemistry Stainning 

Paraffin wax tissue sections of testes were dewaxed in xylene, rehydrated through a graded series of ethanol solutions, washed in distilled water and phosphate buffer saline (PBS). The essential techniques of the IHC assay consisted of repairing the antigen in 0.1 M boiling citrate buffer (pH 6.0) in a pressure cooker; blocking endogenous peroxidase 3% hydrogen peroxide; blocking nonspecific protein binding with 10% goat plasma; incubated overnight at 4 °C with the primary antibodies Cleaved Caspase-3 (1:200; 341034; Zen BioScience, Chengdu, Sichuan, China) and then biotinylated goat anti-rabbit immunoglobulin G (IgG) secondary antibody(1:100; SA2002; Boster, Wuhan, China) was applied to each slide, followed by incubation with strept avidin-biotin complex (SABC; Boster, Wuhan, China) for 30 min at 37 °C. Immunoreaction products were stained with using diaminobenzidine (DAB) (AR1022; Boster, Wuhan, China) as a chromogen. Each slide was counterstained with Mayer’s haematoxylin. Negative controls were prepared with the sections stained without the primary antibody. For each section, seminiferous tubules (200× magnification, Nikon DS-Ri1, Japan) were counted for protein expression levels by integrated optical density (IOD).

### 4.8. Detection of mRNA Expression in the Testis by Quantitative Realtime PCR

Total RNA was extracted from the testis tissue samples with Total RNA Isolation Kit (RE-03011; Foregene, Chengdu, China). Next, RNA was subjected to reverse transcription with a RT-PCR easyTM I Kit (RT-02011) in accordance with the manufacturer’s instructions (Foregene, China). The cDNA product was used as a template for quantitative real time PCR (qRT-PCR) analysis. Sequences for target genes were obtained from the NCBI database. Oligonucleotide primers were designed by use of Primer 5 software and synthesized at Takara (Dalian, China), as shown in Table 1. All qRT-PCR were performed by use of the SYBR® Premix Ex TaqTM II system (DRR820A, Takara, Japan) with on a Bio-Rad iQ5 system, and the relative gene expression was normalized to internal control as β-Actin. Primer sequences for SYBR Green probes of the target genes are described in Table 1.

### 4.9. Western Blotting

For standard western blotting, 0.02 g testicular tissues were homogenized in tissue lysate (P0013G; Beyotime, China) comprising a mixture of phosphatase and protease Inhibitors, followed by centrifugation at 12,000 rpm for 15 min after incubation for 20 min at 4 °C. BCA protein assay kit (P0012S; Beyotime, China) measured supernatants concentration. The supernatant were added to SDS-PAGE loading buffer (100 mM Tris, pH 6.8, 20% glycerol, 200 mM DTT, 4% SDS, 0.03% bromophenol blue) and boiled for 10 min at 100 °C. The protein samples (20 μg each) in loading buffer were subjected to electrophoresis on 12% SDS-polyacrylamide gel (Bio-Rad) for 1.5 h. Then the gel was transferred onto electrophoretically a PVDF membrane (IPVH00010; Millipore, Billerica, MA, USA). The membranes were blocked with 5% nonfat powdered milk in TBST for 1 h and were then incubated with specific primary antibodies including anti-Nrf2 Rabbit pAb (1:1000; 340675, Zen BioScience, Chengdu, Sichuan, China), anti-caspase-3 p17 (Cleaved-Asp175) rabbit pAb (1:1000; 341034; Zen BioScience, Chengdu, Sichuan, China), anti-BAX rabbit pAb (1:1000; 515633, Zen BioScience, Chengdu, Sichuan, China), HO-1/HOMX1 rabbit polyclonal antibody (1:1000; 10701-1-AP, proteintech, wuhan, China), anti-BCL2 antibody (1:200; BA0412, Boster, Wuhan, China ), anti-β-actin (ACTB) antibody (1:1000; BM0627, Boster, Wuhan, China) overnight at 4 °C. The membranes were subsequently incubated with horseradish peroxidase-conjugated anti-rabbit or anti-mouse IgG (1:5000; BA1054 or BA1056, Boster, Wuhan, China) for 1 h at 37 °C. Protein bands were detected using an enhanced chemiluminescence (ECL) system (1708265, Bio-Rad, California, America).

### 4.10. Statistical Analysis

All data were expressed as mean ± standard deviation (X ± SD). Student’s t-test and one-way ANOVA test were performed for all statistical significance analysis using SPSS 22.0 (SPSS Inc., Chicago, IL, USA). A P-value of 0.05 was considered statistically significant.

## Figures and Tables

**Table 1:** Primer sequences used in real-time RT-PCR.

Gene	Primer Sequences (5′-3′)		Length (bp)
	Forward	Reverse	
Nrf2	GAGACGGCCATGACTGAT	TGTGGAACATCTGGTAGACGGC	196
HO-1	CTGGAATGGAAGGAGATGCC	TCAGAACAGCCGCCTCTACCG	132
GSH-Px1	CAGTTCGGACATCAGGAGAAT	AGAGCGGGTGAGCCTTCT	139
Sod2	AAAGGAGAGTTGCTGGAGGC	TGATTAGAGCAGGCGGCAAT	161
Sod3	TTGTTCTGCAACCTGCTACTGG	AGTGCGTGTCGCCTATCTTCTC	124
Caspase-3	CGGACCTGTGGACCTGAAAA	CGGCCTCCACTGGTATCTTC	182
Bcl-2	GGCATCTTCTCCTTCCAGCC	CGACGAGAGAAGTCATCCCC	173
BAX	TGGCGATGAACTGGACAACA	CCCAGTTGAAGTTGCCGTCT	125
β-actin	ACGGTCAGGTCATCACTATCG	GGCATAGAGGTCTTTACGGATG	155

Primer biosynthesis by Sangon Biotech (Shanghai) Co., Ltd.
